# Biomimetic Vascularized iPSC‐Hepatocyte Spheroids for Liver Regeneration

**DOI:** 10.1002/advs.202405662

**Published:** 2024-12-24

**Authors:** Jinglin Wang, Danqing Huang, Haozhen Ren, Yuanjin Zhao

**Affiliations:** ^1^ Division of Hepatobiliary and Transplantation Surgery Department of General Surgery Nanjing Drum Tower Hospital The Affiliated Hospital of Nanjing University Medical School Nanjing 210008 China; ^2^ State Key Laboratory of Bioelectronics School of Biological Science and Medical Engineering Southeast University Nanjing 210096 China

**Keywords:** Biomimetic, liver regeneration, microcapsule, microfluidic, vascularized spheroids

## Abstract

Human induced pluripotent stem cell derived hepatocytes (hiPSC‐heps) hold promising value for acute liver failure (ALF) treatment, while their therapeutic efficacy is usually limited by low cell bioactivity and untargeted in vivo accumulation. Here, inspired by vascularity supporting cellular architectures in the tissues and organs, a novel vascularized hiPSC‐heps spheroid based on microfluidic microcapsules is presented for liver repair via orthotopic transplantation. The microcapsules are comprised of aqueous cores that facilitate hiPSC‐hep aggregating into spheroids, and hybrid hydrogel shells of sodium alginate and hyaluronic acid methacryloyl (HAMA). By selectively degrading the alginate, the microcapsules are imparted with porous HAMA shells, which not only allowed human umbilical vein endothelial cells (HUVECs) to attach and form vascularized networks, but also facilitated communication between HUVECs and hiPSC‐heps. The specific spatial distributions of these cells in the vascularized hiPSC‐hep spheroids can provide nutrition support, promote the hepatic functions, and avoid immune cell attacks. Based on these features, it is illustrated that the vascularized hiPSC‐hep spheroids can repair the acute failing liver more effectively, indicating their practical values in clinical liver repair.

## Graphical abstract

This study introduces biomimetic vascularized microcapsules for delivering hiPSC‐hep spheroids in acute liver failure (ALF). Employing microfluidic electrospray technology, the microcapsules enhance substance exchange and protect spheroids. Animal experiments demonstrate significant liver regeneration, underscoring their potential to improve hepatic function and support vascular system recovery.

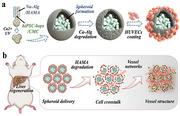

## 1 Introduction

Acute liver failure (ALF) is a critically life‐threatening disease caused by extensive hepatocytes disfunction.^[^ ^1^, ^2^, ^3^, ^4^ ^]^ Numerous therapeutic approaches have been utilized to treat ALF, in which cell transplantation is the most cutting‐edge strategy.^[^ ^5^, ^6^, ^7^ ^]^ It can realize liver regeneration and repairment with the assistance of external cells, including stem cells, hepatic primary/progenitor cells, human induced pluripotent stem cell derived hepatocytes (hiPSC‐heps), and so on.^[^ ^8^, ^9^, ^10^ ^]^ Among these external cells, hiPSC‐heps have been considered as promising cell sources for ALF therapy because of their convenient induction strategy, excellent hepatic function and definite efficacy.^[^ ^11^, ^12^, ^13^ ^]^ Despite with these advantages, the hiPSC‐heps through intravenously injection often get attacked by host immune cells, leading to their cell loss, function damage, and untargeted aggregation.^[^ ^14^, ^15^ ^]^ In addition, the scattered hiPSC‐heps that infiltrate the liver struggle to optimally perform therapeutic functions such as mitigating damage, augmenting proliferation, and facilitating hepatic rejuvenation, given their challenges in cultivating a conducive liver microenvironment.^[^ ^16^, ^17^ ^]^ Moreover, the ischemic, hypoxic, or inflammatory conditions of the impaired liver can severely affect the viability of these transplanted hiPSC‐heps.^[^ ^7^, ^18^ ^]^ Therefore, new cell delivery strategies are still desired to improve the survival and functionality of the hiPSC‐heps for the ALF treatment.

**Figure 1:**
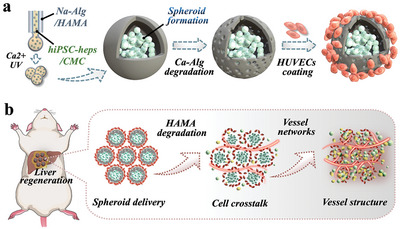
Schematic illustration of the biomimetic vascularized hiPSC‐hep spheroids. a) Schemes of the microfluidic fabrication and culture process of the biomimetic vascularized microcapsule. b) The obtained microcapsules are assembly implanted into ALF rat for liver regeneration.

Here, inspired by vascularity supporting cellular architectures in our tissues and organs,^[^ ^19^, ^20^ ^]^ we proposed a novel vascularized hiPSC‐heps spheroid for liver repair via orthotopic transplantation, as schemed in **Figure** **1**. Compared with dispersed cells, the cell‐to‐cell interactions within cell spheroid improve cell communications, contributing to the establishment of biomimic structure and microenvironment.^[^ ^21^, ^22^ ^]^ Besides, in the presence of vascularized structures, the albumin secretion of cell spheroids can be greatly improved.^[^ ^23^ ^]^ However, due to the lack of high‐precision and controllable preparation methods, traditional cell spheroids usually suffer from several drawbacks, such as inaccurate size and inhomogeneous structure.^[^ ^24^, ^25^ ^]^ Additionally, cell spheroids tend to stick together during local implantation, resulting in nutrient deficiencies. Furthermore, the efficacy of cell spheroids in liver regeneration therapy remains to be explored. Thus, the development of an efficient stratagem for achieving vascularized cell spheroids is important for hiPSC‐heps facilitated liver repair.

In this paper, we employed a simple microfluidic electrospray microcapsule technique to fabricate the desired vascularized hiPSC‐hep spheroids for ALF treatment. The microfluidic microcapsules were with a carboxymethyl cellulose (CMC) core loaded with hiPSC‐heps, and a hybrid‐hydrogel shell of alginate and hyaluronic acid methacryloyl (HAMA). The precise fluid manipulation of microfluidics imparted the microcapsules with exceptional uniformity, stability, and dimensional controllability, allowing the hiPSC‐heps assembling into spheroids. After selectively degrading alginate, the microcapsules with porous HAMA shell structures could be obtained, which not only allowed human umbilical vein endothelial cells (HUVECs) to attach, but also facilitated communication between HUVECs and hiPSC‐heps. It was demonstrated that the interactions between hiPSC‐heps can promote the hepatic functions. Besides, the surrounded HUVECs network provided the hiPSC‐hep spheroids with a protective barrier to avoid immune cell attack and support nutrition. Thus, this vascularized hiPSC‐hep spheroids can repair the acute failing liver more effectively. These results indicated that the proposed biomimetic vascularized hiPSC‐hep spheroids are remarkably promising for clinic liver regeneration.

## 2 Results

**Figure 2:**
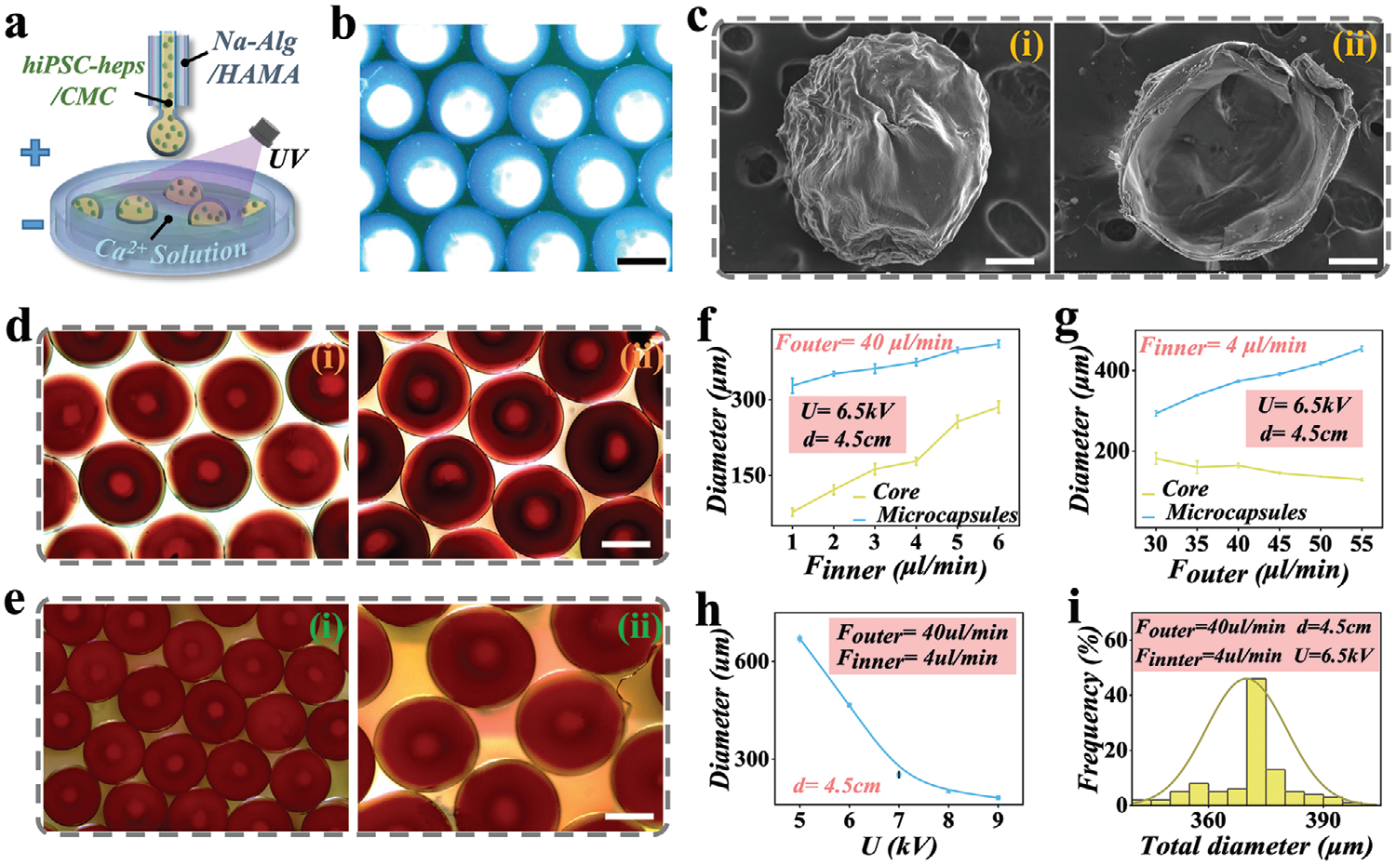
Preparation and regulation of the CMC/Ca‐Alg‐HAMA microcapsules. a) Schematics of the microfluidic fabrication process of the microgels, scale bar is 100 µm. b) The microfluidic microcapsules observed using a stereomicroscope. c) SEM images of the whole microcapsule (i), the interior of microcapsules (ii), scale bar is 200 µm, respectively. d,e) Optical microscope images of microcapsules with different Fouter/ Finner, scale bar is 200 µm, respectively. f–i) Overall size and core size distributions of the CMC/Ca‐Alg‐HAMA microcapsules under the different parameters. U referred to as voltage, and d referred to as acquisition distance.

To generate microcapsules with high uniformity, we fabricated a single emulsion capillary chip with coaxial geometry and utilized microfluidic electrospray technology (Figure S1, Supporting Information). The internal phase solution consisted of a CMC aqueous solution, which featured with excellent cell compatibility, while the external phase consisted of a mixed solution of HAMA and high viscosity sodium alginate (Na‐Alg). The shell pregel solution was infused into the external channel, while the core pregel solution was infused into the internal channel of the single emulsion microfluidic device, respectively, as schemed in **Figure** **2**a. According to the coaxially assembled microfluidic channels, the CMC flow can be enveloped by the Na‐Alg/HAMA flow. Under the influence of electrostatic forces, the two pre‐hydrogel flows were induced to overcome surface tension before being cut into droplets with core‐shell structures at the outlet of the device. The Na‐Alg/HAMA shell then dropped into the calcium chloride collection pool.^[^ ^26^, ^27^ ^]^ The Na‐Alg component rapidly reacted with calcium ions, forming solid calcium alginate (Ca‐Alg); meanwhile, we applied ultra‐violet (UV) light continuously illuminated the collection pool to polymerize HAMA component. Finally, microcapsules with solidified Ca‐Alg/HAMA shell can be obtained (Figure 2b). The rapid ionic crosslinking of Na‐Alg allowed the internal CMC hydrogel being confined within the microcapsules, providing a limited space for subsequent cell culture. Furthermore, as shown in Figure 2c, the core‐shell structure of a single CMC/Ca‐Alg‐HAMA microcapsule was confirmed by scanning electron microscopy (SEM).

Owing to the exceptional operability of microfluidic electrospray technology, we investigated the correlation between the flow velocity of the internal (Finner) and outer (Fouter) phases and the diameter of the core‐shell microcapsules (Figure 2d). It can be found that when the Finner increased, the core diameter would increase, and the shell thickness would decrease simultaneously (Figure 2d(i,ii),f). Conversely, an increase in the Fouter resulted in an increase in the diameter of the microcapsule and shell thickness, while the diameter of the core layer decreased (Figure 2e(i,ii),g). Additionally, we discovered that the diameter of the microcapsules decreased with an increase in voltage (Figure 2h). By dynamically adjusting the flow rates, acquisition distance, and voltage, we ultimately determined that the Finner was 4 µL min^−1^, the Fouter was 40 µL min^−1^, the voltage was 6.5 kV, and the height was 4.5 cm, and the resulting microspheres exhibited excellent monodispersity, as shown in Figure 2i.

**Figure 3:**
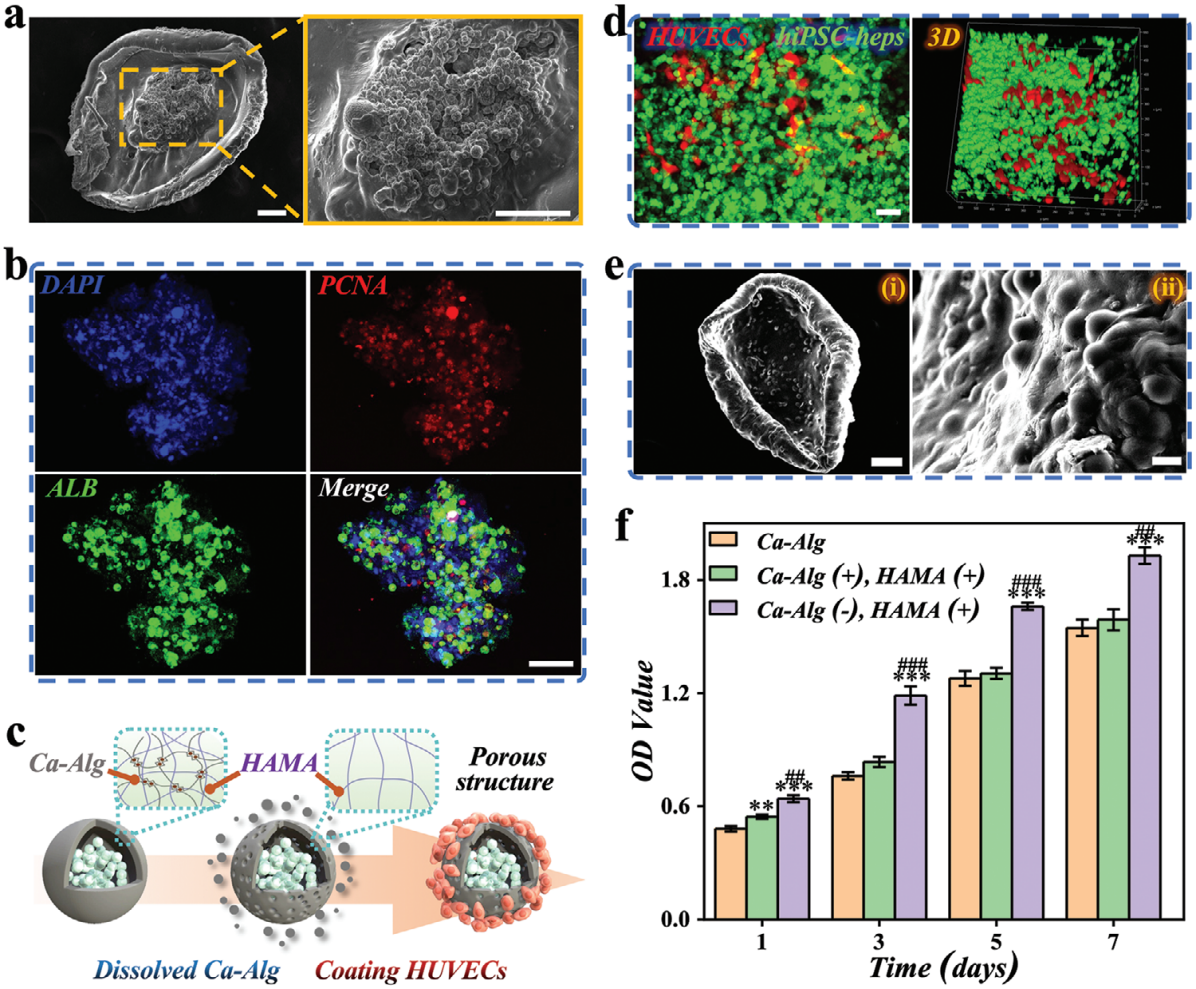
Generation and characterization of porous vascularized microcapsules. a) SEM images of the interior of microcapsules with hiPSC‐hep spheroids encapsulated, scale bars are 50 µm. b) Laser scanning confocal microscopy (LSCM) image of a hiPSC‐hep spheroid‐encapsulated microcapsule with ALB/PCNA staining, scale bar is 50 µm. c) Schemes of the selective degradation and HUVECs coating process of the microcapsules. d) LSCM of the communications between hiPSC‐heps and HUVECs, scale bar is 50 µm. e) SEM images of the interior (i) and surface (ii) of porous vascularized microcapsules with hiPSC‐hep spheroids encapsulated, scale bars are 50 µm in (i) and 10 µm in (ii). f) The viability of hiPSC‐hep spheroids encapsulated in three different hydrogel shells. All data are presented as means ± SE (n = 3). ^*^ *p* < 0.05; ^***^ *p* < 0.001 (compared with Ca‐Alg). ^##^ *p* < 0.01; ^###^ *p* < 0.001 (compared with Ca‐Alg (+), HAMA(+)).

Microcapsules with a core‐shell structure offer a versatile system for nutrient circulation and biomolecular exchange for encapsulated cells, while also providing protection against larger immune cells and antibodies. In this study, we investigated the potential of using core‐shell microcapsules as 3D carriers for hiPSC‐heps culturation. Currently, using hiPSC lines is a potential strategy for generating hepatocyte‐like cells. These lines have the unique ability to self‐renew consistently and indefinitely in vitro, making them an ideal cell source for producing functional cell types. Moreover, the elimination of genomic integration and background oncogenic transgene expression in hiPSC‐derived cells has enhanced their safety and potential for clinical applications in cell therapy. The experimental process involved mixing the suspension of hiPSC‐heps with CMC solution, followed by microfluidic electrospray process to obtain microcapsules loaded with hiPSC‐heps. Considering that the cell viability rate enclosed within the voltage exceeding 10 kV falls below 90%, we have opted to utilize voltages lower than 10 kV (Figure S2, Supporting Information). The encapsulated cells were then cultured in a cell culture medium, and exhibited good vitality and proliferation (Figures S3 and S4, Supporting Information). As shown in **Figure** **3**a, SEM analysis revealed that the hiPSC‐heps were clustered in the center of the core‐shell and closely connected with each other to form spheroids. The assessment of liver function expression levels also revealed albumin (ALB) expression and cell proliferation capability (Figure 3b; Figure S5a, Supporting Information). When compared to 2D culture, hepatocytes organized in a core‐shell structure demonstrated superior ALB secretion and the proliferating cell nuclear antigen (PCNA) expression (Figure S5b—d, Supporting Information). This may be attributed to the excellent biological function of the microcapsule hydrogel, which promotes hiPSC‐hep spheroids formation.

The liver is an organ that possesses a highly intricate vascular network, playing a critical role in maintaining internal environmental homeostasis, facilitating nutrient and gas exchange, and eliminating metabolic waste products.^[^ ^28^, ^29^ ^]^ However, due to the limited diffusion of oxygen and nutrients, cells located at a distance from the capillaries often encounter hypoxic and apoptotic conditions.^[^ ^30^ ^]^ To address this issue, vascularized hiPSC‐hep spheroids have been developed. Since the gaps between Ca‐Alg hydrogels are restricted, HAMA hydrogel exhibits a comparatively less compact structure characterized by larger interstitial spaces, which facilitates dialogue between cells. Then, we selectively degraded Ca‐Alg using sodium citrate while the HAMA hydrogel maintained the shell structure of microcapsules, and subsequently adhered HUVECs to the surface of the HAMA shell, as depicted in Figure 3c. Compared with the Ca‐Alg‐degraded HAMA hydrogel, we can hardly observe porous structures on the surface of the hybrid Ca‐Alg/HAMA hydrogel. However, upon degrading Ca‐Alg and leaving only HAMA, the pore size in the gel shell increased to over 10 µm, which was sufficient for effective substance exchange between cells (Figure S6a, Supporting Information). We also discovered that by altering the concentration of, it is possible to the pore size of the hydrogel formed after Alg degradation. When Alg is at a concentration of 1 wt.% and HAMA is at 2 wt.%, the pore size is ≈15 µm, which yields optimal cellular activity (Figure S6b, Supporting Information). Furthermore, we have also simulated the degradation of the hydrogel in vitro, and on the seventh day, it can still maintain 50% of its initial mass (Figure S7, Supporting Information). This indicated that the functionality provided by the hydrogel can sustain the recovery process of liver failure.

We also utilized laser scanning confocal microscopy (LSCM) to observe the internal and external adhesion of HAMA‐only shells of hiPSC‐heps with green fluorescence and HUVECs with red fluorescence (Figure 3d). Our observations revealed that hiPSC‐heps were able to penetrate the shell and communicate spatially with HUVECs, which could potentially enhance cell function. This LSCM phenomenon was further confirmed by SEM results. On the shell of the hydrogel, not only did HUVECs adhere tightly to the outer layer (Figure 3e(i)), but a large number of hiPSC‐heps also adhered firmly to the inner layer of the shell (Figure 3e(ii)). The dimensions of both the core and shell were also ascertained in the vascularized hiPSC‐hep spheroids (Figure S8, Supporting Information). The diameter of the core in the hiPSC‐Hep spheroids was measured at 219.708 ± 9.707 µm, whereas the diameter of the shell was recorded at 358.810 ± 5.409 µm. In order to visualize vascularization within the microcapsules, LAMININ, a prominent basement membrane glycoprotein of blood vessels, was utilized for staining the coated HUVECs,^[^ ^31^ ^]^ as shown in Figure S9 (Supporting Information). This revealed the emergence of certain tubular connections, which may facilitate effective communication between hiPSC‐heps and HUVECs, thereby mimicking the physiological heterogeneous structure and cellular microenvironment of the liver.

Previous studies have shown that vascularized tissues can significantly enhance the microenvironment of the cell spheroids and improve cell function.^[^ ^19^ ^]^ Therefore, we conducted cell counting kit 8 (CCK8) activity experiments to test the effect of shell hydrogels with different compositions on the function of the hiPSC‐hep spheroids. Our findings indicated that, compared with Ca‐Alg shell and Ca‐Alg‐HAMA shell, HAMA hydrogel shell with degraded Ca‐Alg were able to maintain the activity of hiPSC‐hep spheroids better (Figure 3f). Moreover, we conducted an examination of the ALB, urea, and cytochrome P450 (CYP) activity secretions from the vascularized hiPSC‐hep spheroids. We discovered that, compared to the only hiPSC‐hep spheroids, the functionality within the co‐culture structure was enhanced (Figure S10, Supporting Information). This suggested that hiPSC‐heps can effectively communicate with HUVECs through a large enough hydrogel gap, which was of importance in enhancing the function of hiPSC‐hep spheroids. In summary, cell‐cell interactions and efficient vascular networks are crucial in promoting the function of hiPSC‐hep spheroids.

**Figure 4:**
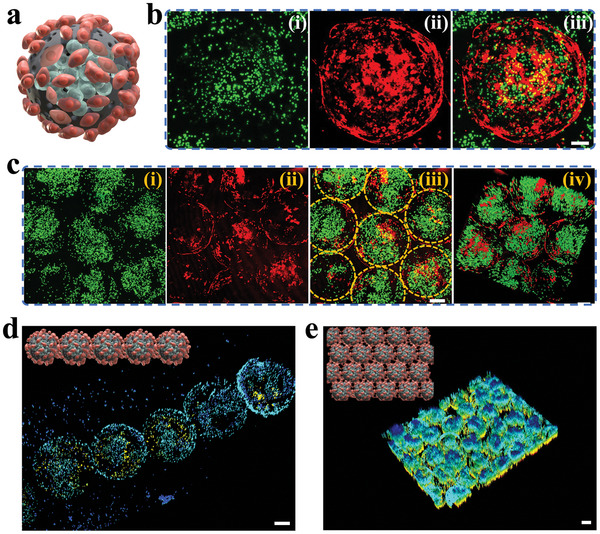
The well‐organized vascular network of the vascularized microcapsules. a) Schemes of the vascularized microcapsules with hiPSC‐hep spheroids encapsulated and HUVECs coating. b) LSCM image of single microcapsule with hiPSC‐hep spheroid (green) encapsulated and HUVECs (red), scale bar is 50 µm. c) Close assembly of vascularized microcapsules by HUVECs connections, scale bars are 100 µm. d,e) Single‐row (d) and sheet‐like (e) assembly of the vascularized microcapsules, scale bars are 100 µm.

By following the aforementioned procedure, we successfully obtained vascularized core‐shell microcapsules that were loaded with hiPSC‐hep spheroids. The individual microcapsule was schematically represented in **Figure** **4**a. To further observe the 3D structure of the spatial distribution of the two types of cells in the microcapsule, we performed LSCM observations and reconstructed them. The hiPSC‐heps and HUVECs were found to be located between the inner and outer parts of the hydrogel shell, and the boundary was clearly visible (Figure 4b). Additionally, the red HUVECs adhered to the outer surface of the shell, forming a spherical circle, while the hiPSC‐heps wrapped inside were clustered. Some of the hiPSC‐heps were scattered on the surface of the shell or penetrated the microcarrier. This indicated that the shell effectively enveloped the hiPSC‐hep spheroids, while also allowing for the migration of hepatocytes through pores. This would not affect the function of the spheroids in following in vivo ALF experiment.

Surprisingly, the vascularized microcapsules that we prepared may have self‐assembled due to the signals sent by HUVECs. Compared to unvascularized hiPSC‐hep spheroids, vascularized hiPSC‐hep spheroids microcapsules released a greater amount of vascular endothelial growth factor (VEGF) (Figure S11a, Supporting Information). Furthermore, the expression of phosphorylated VEGFR2 on HUVECs in vascularized microcapsules with hiPSC‐hep spheroids was increased compared to those hiPSC‐hep spheroids, and the expression of ZO‐1 was also elevated (Figure S11b,c, Supporting Information). The presence of hiPSC‐hep spheroids enhanced the tight communication of HUVECs. These microcapsules were distributed in a staggered manner, forming a tight connection of hexagonal stacking on the plane, as shown in Figure 4c(i‐iii). Furthermore, due to the effective packaging of HUVECs, the hiPSC‐heps spheroid in the center of the microcapsules would not stack with each other to form an aggregated state, and each microcapsule would ensure effective nutrient supply for the hiPSC‐heps spheroid. By 3D reconstruction, each microcapsule presented a fully spherical shape, and the shells of the HUVECs did not collapse (Figure 4c(iv)). The hexagonal prismatic shape of the biomimetic liver lobule also sets the stage for the function of the self‐assembled microcapsules. Subsequently, we found that if multiple microcapsules are arranged in a straight line, after a period of culture, the HUVECs contacting between two adjacent microcapsules will adhere to each other, thus forming a single‐row beaded structure, and the microcapsules cannot be easily dispersed (Figure 4d). Therefore, we further assembled these microcapsules into sheet‐like tissues. We found that hexagonally tightly packed microcapsules were uniformly arranged to form a good plane, and the microcapsules were tightly connected and cannot be easily dispersed, indicating promising applications in tissue engineering (Figure 4e).

**Figure 5:**
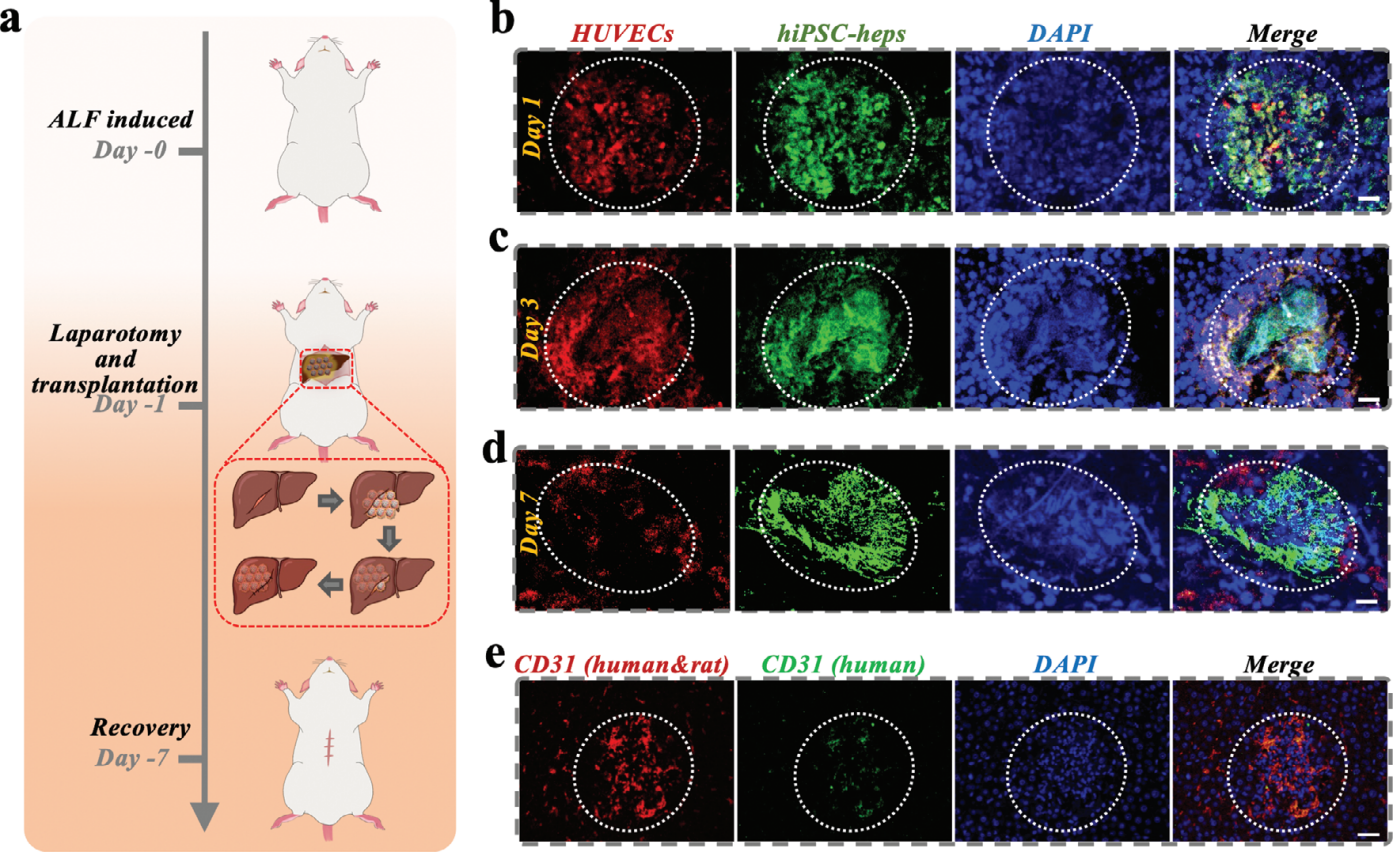
In vivo investigations evaluating neovascularization and targeted delivery capabilities. a) Conceptual depiction of ALF rats undergoing treatment with the assembled, vascularized microcapsules. b–d) LSCM visualization of vascular networks, with HUVECs identified by mCheery (red), hiPSC‐heps identified by GFP (green), and nuclei stained with DAPI (blue). Scale bars represent 50 µm on days 1, 3, and 7. e) LSCM representation employed to signify endothelial cells via CD31, with red markers highlighting HUVECs and rat endothelial cells, and green markers pinpointing HUVECs. Scale bar corresponds to 50 µm.

Based on the above advantages, vascularized hiPSC‐hep spheroids were then employed for treating liver diseases. To showcase the viability of this method, we created ALF rat models by injecting D‐galactosamine (D‐Gal) intraperitoneally. The damaged liver was then treated with vascularized hiPSC‐hep spheroids, as illustrated in **Figure** **5**a. An incision of one centimeter was made in the upper abdomen of the rat to expose the liver, and multiple transplants of vascularized microcapsules were performed beneath the liver capsule. To assess the in vivo colonization of the microcapsules, we using GFP to label hiPSC‐ hep spheroids and mCherry to label HUVECs, allowing us to observe the transplantation status of microcapsules in the liver using immunofluorescence. After staining the frozen liver sections with DAPI, we can identify the cell aggregates with green fluorescence as hiPSC‐ hep spheroids. On the first day after transplantation, hiPSC‐hep spheroids were completely enveloped by an annular structure formed by HUVECs (Figure 5b). Over time, the hiPSC‐heps gradually spread out and showed a tendency to penetrate the HUVECs' network. By the seventh day, the network structure of HUVECs remained intact, with dense connections between cells, but the inner hiPSC‐heps had penetrated and dispersed throughout the liver (Figure 5c‐d). Additionally, by using specific anti‐human CD31 antibodies (green fluorescence labeling HUVECs on the transplanted microcapsules) and anti‐human and anti‐rat CD31 antibodies (red fluorescence labeling both HUVECs on the transplanted microcapsules and endothelial cells in the rat), we found that the green fluorescence overlapped with the red fluorescence, with areas of non‐overlap indicating the formation of the vascular network (Figure 5e).

**Figure 6:**
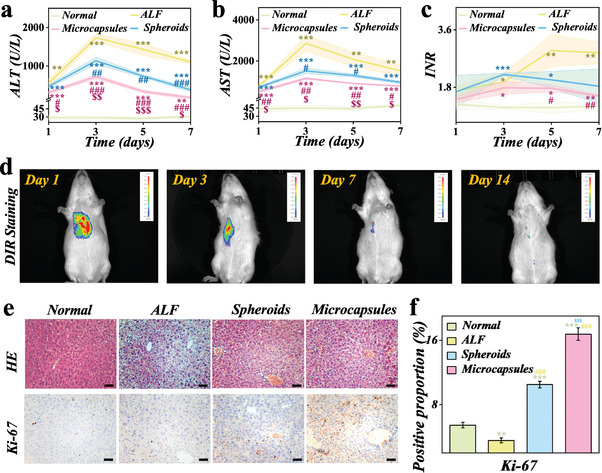
The in vivo therapeutic effect of vascularized microcapsules on ALF rat models. a–c) Evaluation of the ALT, AST, and INR levels of ALF rats from different groups. d) The location and intensity of DIR‐stained hiPSC‐heps in ALF rats detected by small animal imaging system. e) HE and Ki‐67 staining images of different groups, scale bars are 50 µm. All images presented in the results are representative of at least 3 images per liver. f) The positive proportion of Ki‐67 staining in three groups. All data are presented as means ± SE (n = 3). ^*^ *p* < 0.05; ^**^ *p* < 0.01; ^***^ *p* < 0.001 (compared with Normal). ^#^ *p* < 0.05; ^##^ *p* < 0.01; ^###^ *p* < 0.001 (compared with ALF). ^$^ *p* < 0.05; ^$$^ *p* < 0.01; ^$$$^ *p* < 0.001 (compared with Spheroids).

To gain further insight into the therapeutic effects of biomimetic vascularized hiPSC‐heps spheroids in the treatment of ALF, we conducted a study involving 60 ALF rats. The efficacy of vascularized hiPSC‐hep spheroids was observed as follows: the first group underwent sham operation (ALF group), the second group received hiPSC‐hep spheroids transplantation (Spheroids group), and the third group received transplantation of vascularized hiPSC‐hep spheroid microcapsules (Microcapsules group), non ALF rats were included as control (Normal group). The total number of hiPSC‐heps in the second and third groups was 10^7^. Subsequently, we proceeded to test the indexes of liver function, coagulation, and blood ammonia (NH_3_) in each group. The detected indicators encompass alanine aminotransferase (ALT) for assessing the extent of hepatocellular damage, aspartate aminotransferase (AST) for evaluating liver cell injury, international normalized ratio (INR) to measure the degree of coagulation impairment, and NH_3_ to determine if there are any abnormalities in liver metabolism. It revealed that the above indexes and survival rate in the two groups of rats that underwent hiPSC‐heps transplantation were significantly better than those in the ALF group (**Figure** **6**a‐c; Figures S12 and S13, Supporting Information). These results indicated that the liver failure can be controlled and the liver damage can be successfully treated using hiPSC‐heps transplantation. Furthermore, compared with ALF and Spheroids group, the degree of damage in each index was reduced in the Microcapsules group and the recovery was faster.

To further investigate the colonization of microcapsules in the liver, we utilized fluorescent cell membrane dyes as markers to dye them before transplantation, and then conducted small animal in vivo imaging after transplantation, as shown in Figure 6d. Our results showed that the transplanted microcapsules can be concentrated in the liver area as well as exhibiting considerable fluorescence intensity, without transferring to other parts of the body. Additionally, the microcapsules can maintain an ideal fluorescence intensity for up to 7 days, indicating that they maintained their functions in the liver. Furthermore, we performed dynamic analysis of the liver in vivo using the IVIM's All‐in‐One Intravital Microscopy System, a fully integrated live confocal/two‐photon microscopy system. By labeling blood flow with Evans Blue (pseudocolored red) and HUVECs on the microcapsules with GFP, we observed the assembly of blood vessels in the rat liver after the microcapsules transplantation (Figure S14, Supporting Information). The matching fluorescence between the perfusion of the rat liver and the transplanted HUVECs confirms the formation of a new vascular network after transplantation.

We also observed the cell state inside the liver by staining liver tissue sections. We conducted hematoxylin‐eosin (HE) staining to observe the pathological changes of liver tissue. The Spheroids group exhibited slightly more severe congestion and vacuolation than the Microcapsules group (Figure 6e). Moreover, Ki‐67 staining was used to detect the degree of cell proliferation. Compared to the other two groups, the microcapsules group demonstrated significantly improved hepatocyte proliferation (Figure 6f). Furthermore, the efficacy of microcapsules in promoting liver regeneration, inhibiting liver apoptosis, and modulating inflammatory responses has been further validated through western blotting analysis. We discovered a increase in the expression of the liver regeneration protein, yes‐associated protein 1 (YAP), and PCNA in the microcapsules group. Concurrently, the expression of the apoptosis‐inhibiting protein, B‐cell lymphoma‐2 (Bcl2), also notably increased, while the expression of the apoptosis‐promoting protein, Bcl2‐associated X (Bax), was suppressed (Figure S15, Supporting Information). Moreover, the expression of the inflammatory factors IL‐1β and IL‐6 in the microcapsules group notably declined (Figure S16a,b, Supporting Information). We also examined the expression levels of ALB, urea, and CYP activitives in the liver post‐transplantation, discovering that the transplantation of microcapsules aids in the restoration of liver function (Figures S16c,d and S17, Supporting Information). These results suggested that our biomimetic vascularized microcapsules can effectively promote liver repair, possibly due to the formation of vascular networks. Moreover, we performed staining on the infiltrating lymphocytes surrounding the graft post transplantation. We observed a pronounced increase in CD4+ T cells (red), CD8+ T cells (orange), IL‐17+ T cells (Th17, pink), and FOXP3+ T cells (Treg, green) around the spheroids group without HUVECs encapsulation, compared to the microcapsules group with HUVECs encapsulation (Figure S18, Supporting Information). This suggests that HUVECs encapsulation can effectively mitigate rejection responses.

## 3 Discussion

In conclusion, we have developed biomimetic vascularized microcapsules for the delivery of hiPSC‐hep spheroids in ALF. The spheroids were encapsulated in hydrogel microcapsules with Ca‐Alg and HAMA shells using microfluidic electrospray technology. This technology allowed for precise control of the size, monodispersity, and composition of the microcapsules, while the selective hydrogel degradation facilitated the formation of porous structures. HUVECs adhered to the surface of the microcapsules, creating a network structure that enabled substance exchange and provides barrier protection for the encapsulated hiPSC‐hep spheroids. This approach allowed for the delivery of hiPSC‐hep spheroids without the risk of clumping, thereby enhancing their therapeutic potential. In animal experiments, we observed liver regeneration after implanting the microcapsules into ALF rats. The vascularized hiPSC‐hep spheroids we constructed not only enhance substance exchange and improve hepatic metabolic capacity, but also aid in situ transplantation, thereby increasing the efficiency of vascular system regeneration.

In developing new cell therapies, maintaining the vitality and functionality of cells is crucial for ensuring the safety and efficacy of treatments. It is also essential to focus on the immune rejection responses in allogeneic cell transplantation, as this will provide guidance for clinical medication.^[^ ^32^ ^]^ In addtion to iPSCs, mesenchymal stem cells are also suitable for cell therapy due to their lack of MHC class II antigens and their ability to secrete anti‐inflammatory factors, making allogeneic cell therapy possible.^[^ ^33^ ^]^ Moreover, significant advancements in cell engineering technologies within the fields of cell and synthetic biology have provided new strategies for protecting and prolonging the lifespan of genetically engineered cells in vivo. These strategies include the use of biomaterials and proteins to develop adjustable drug delivery systems. For instance, cells can be encapsulated in lipid particles, which not only enhance drug delivery effectiveness but also release immunomodulatory drugs that induce tolerance, thereby extending the survival time of grafts.^[^ ^34^, ^35^ ^]^ Furthermore, the liver microenvironment is characterized by a multitude of cell types, high density, and relatively distinct zones. Consequently, our future work will involve constructing complex vascularized liver tissues that incorporate interactions between multiple cellular components, providing a framework for cell transplantation in tissue engineering.

## 4 Experimental Section

### Fabrication of the Microfluidic Device

A single emulsion microfluidic chip was fabricated using two capillaries and a glass slide. The inner and outer capillaries were positioned coaxially, with diameters of 100 and 300 µm, respectively.

### Cell Culture

The hiPSCs were got from the Clinical Stem Cell Center at Nanjing Drum Tower Hospital. The cells were nurtured in a six‐well plate (5 × 10^5^ cells per well) utilizing mTeSR1 medium combined with Matrigel under conditions of 5% CO_2_ at 37 °C. Subsequently, the cells were cultured in RPMI1640 medium supplemented with a blend of Activin A, BMP4, bFGF, B27, and Wnt3a for a day, before transitioning to RPMI1640 with a combination of Activin A, BMP4 and bFGF for three days to stimulate definitive endoderm cell development. To instigate hepatoblast formation, the endoderm cells were cultivated in RPMI1640 medium supplemented with KGF, SB431542, and B27 for two days, followed by a change to RPMI1640 medium with KGF, BMP4, BMP2, bFGF, and B27 for three days. In order to facilitate the differentiation into hepatic progenitor cells (HPCs), the hepatoblasts underwent cultivation in DMEM/F12 medium, supplemented with B27, forskolin, SB431542 EGF, CHIR99021, LPA, Dex, and S1P over a period of 6 – 8 days. In order to produce mature hepatocytes (hiPSC‐heps), the HPCs underwent cultivation in Williams’ E medium, complemented with a combination of B27, forskolin and SB431542 for a duration of 21 days.

### Fabrication of the Vascularized hiPSC‐hep Spheroids Microcapsules

To create vascularized hiPSC‐hep spheroid microcapsules, ≈10^7^ hiPSC‐heps cells are dissolved in 1 ml of CMC solution, with the CMC acting as the internal phase at 1.0 wt.%, carrying the hiPSC‐heps, while 1.5 wt.% Na‐Alg (medium viscosity) and 2.0 wt.% HAMA served as the external phase, and the collection pool was 2.0 wt.% CaCl_2_ solution. The coaxially flowed internal and external solutions allowed the outer phase to wrap around the inner phase, meanwhile the electrostatic effect of the electrospray cutting the flow into droplets, followed by collecting the droplets in calcium chloride with 1 min ultra‐violet exposure. By adjusting the voltage, collection distance, internal and external phase flow rates, microcapsules with varying morphologies were obtained. Approximately, microcapsules with an inner phase diameter of 200 µm were obtained. After a 7‐day culture period, the hiPSC‐heps formed spheroids. The microcapsules were then immersed in a 10.0 wt.% sodium citrate solution, which can degrade the Ca‐Alg compartments. HUVECs were cultured in each well to cover the surface of the hiPSC‐hep spheroid microcapsules.

### Cell Transfection

hiPSC‐heps were subjected to transfection with GFP lentivirus (Ubi‐ MCS‐3FLAG‐SV40‐Cherry‐IRES‐puromycin), while HUVECs were transfected with mCherry lentivirus (Ubi‐MCS‐3FLAG‐SV40‐Cherry‐IRES‐puromycin). The objective of introducing the mCherry or GFP lentivirus vector was to label the cells with either a red or fluorescent marker.

### Cell Viability Assay

The viability of hiPSC‐hep spheroids encapsulated in three different hydrogel shells for long‐term culture was assessed using CCK8. The three hydrogel groups were as follows: 1.5 wt.% Ca‐Alg, 1.5 wt.% Ca‐Alg with 2.0 wt.% HAMA, and 1.5 wt.% Ca‐Alg that was dissolved, leaving only 2.0 wt.% HAMA. All procedures were repeated three times.

### DIR Iodide Staining

To visualize the microcapsules, DIR iodide staining was performed. 50 µmol L^−1^ DIR buffer was mixed with the microcapsules, cultured at 37 °C for 20 min following manufacturer's protocol (Fanbo Biochemicals), followed by washing with phosphate‐buffered saline and transplanting into rats. The rats were anesthetized with chloral hydrate and placed in the and images were acquired at days 1, 3, 7, and 14. The 748/780 nm excitation/emission wavelength was observed using a small animal in vivo imaging system (IVIS, AniView 600, Biolight Biotechnology, China). All procedures were repeated three times.

### Animal Experiments

The research adhered strictly to the guidelines established by the Animal Ethics Committee of Drum Tower Hospital, affiliated with the Medical School of Nanjing University (No. 20230401). Sixty Sprague Dawley (SD) rats were administered 0.6 g kg^−1^ D‐Gal to ALF, and were subsequently randomized and divided into three groups of 20 rats each: the sham operation group (ALF group), the hiPSC‐heps spheroids transplantation group (Spheroids group) with a total cell volume of 10^7^, and the vascularized hiPSC‐hep spheroid microcapsules group (Microcapsules group), which had the same cell quantity and proportion as the Spheroids group. Specifically, after anesthetizing the rats, we performed an exploratory laparotomy and located the liver to carry out the microcapsules transplantation under the liver capsule. We divided the transplantation into two areas: the left lobe and the right lobe of the liver. The levels of ALT, AST, INR, and NH_3_ were measured using an automated chemistry analyzer (TBA30‐FR, TOSHIBA, Japan) following treatment. All procedures were repeated three times.

### Immunofluorescence Staining

The frozen sections were fixed using a 4% formalin solution, washed three times, and blocked prior to overnight incubation with primary antibody. Following three additional washes, the sections were incubated with a secondary antibody containing a fluorescent group. After three additional washes, the nuclei were stained and the sections were observed using a fluorescence microscope following sealing. All images presented in the results are representative of at least 3 images per liver.

### HE Staining

The sections were fixed and dehydrated using a series of increasing alcohol concentrations. The transparent tissues were then embedded in wax, cut into thin slices, and placed on a glass slide to dry. Following dewaxing with xylene and staining with HE, the sections were dehydrated using pure alcohol and made transparent with xylene. Finally, the sections were sealed and observed using a microscope. All images presented in the results are representative of at least 3 images per liver.

### Statistical Analysis

All experimental data were analyzed with the Origin 2019b software. The comparisons between data from every two groups were conducted by a two‐sided Student's *t*‐test. *P* < 0.05 was considered significant.

## Conflict of Interest

The authors declare no conflict of interest.

## Author Contributions

Y.J.Z. performed conceptualization. H.Z.R. and J.L.W. performed methodology. J.L.W. and D.Q.H. wrote original draft. H.Z.R. and Y.J.Z. wrote, reviewed, and edited.

## Supporting information



Supporting Information

## Data Availability

Research data are not shared.
