# Supplementary material for: Biomimetic Vascularized iPSC‐Hepatocyte Spheroids for Liver Regeneration
Source: Adv Sci (Weinh). 2024 Dec 24;12(6):2405662. doi: 10.1002/advs.202405662 (PMC11809326; doi:10.1002/advs.202405662)
Supplement: Supplementary file 1 — Supporting Information [file ADVS-12-2405662-s001.docx]

**Biomimetic** **vascularized** **iPSC-hepatocyte spheroids for liver regeneration**

Jinglin Wang^1^, Danqing Huang^1^, Haozhen Ren^1,^*, Yuanjin Zhao^1,2,^*

1 Division of Hepatobiliary and Transplantation Surgery, Department of General Surgery, Nanjing Drum Tower Hospital, The Affiliated Hospital of Nanjing University Medical School, Nanjing, 210008, China

2 State Key Laboratory of Bioelectronics, School of Biological Science and Medical Engineering, Southeast University, Nanjing, 210096, China

*Correspondence: haozhenren@njglyy.com (Haozhen Ren); yjzhao@seu.edu.cn (Yuanjin Zhao)

**Supplementary figures**


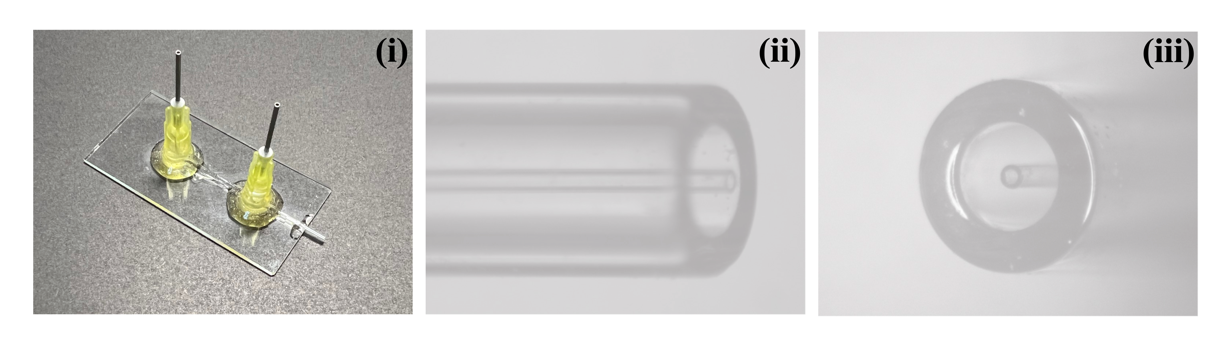


**Figure S1.** (i) The image of a single emulsion microfluidic chip. The stereomicrograph of the capillary assembly (i) and the coincidence of the central axis of the two capillaries (ii).


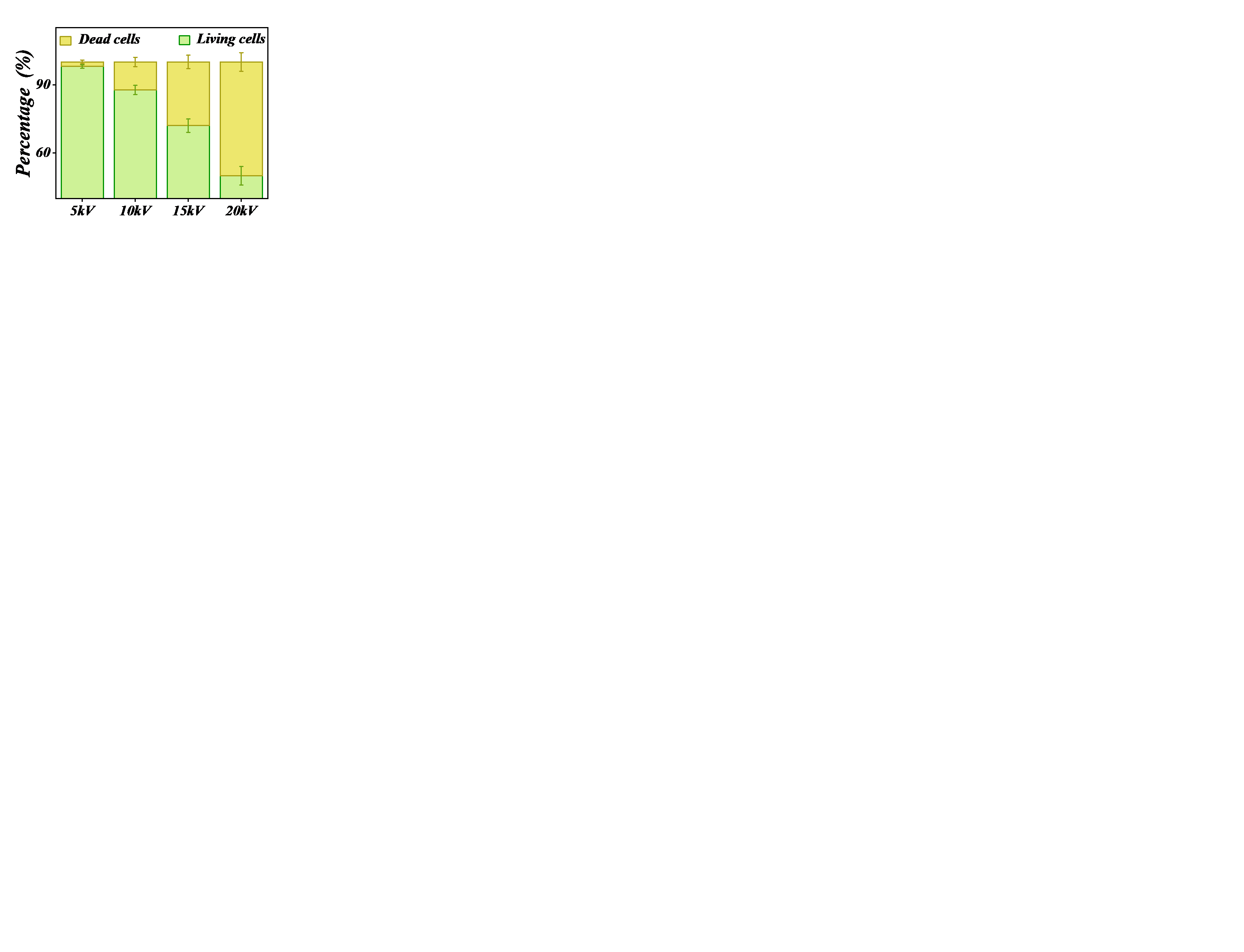


**Figure S2.** The proportion of viable and non-viable cells under different voltage intensities. All data are presented as means ± SE (n = 3).

**
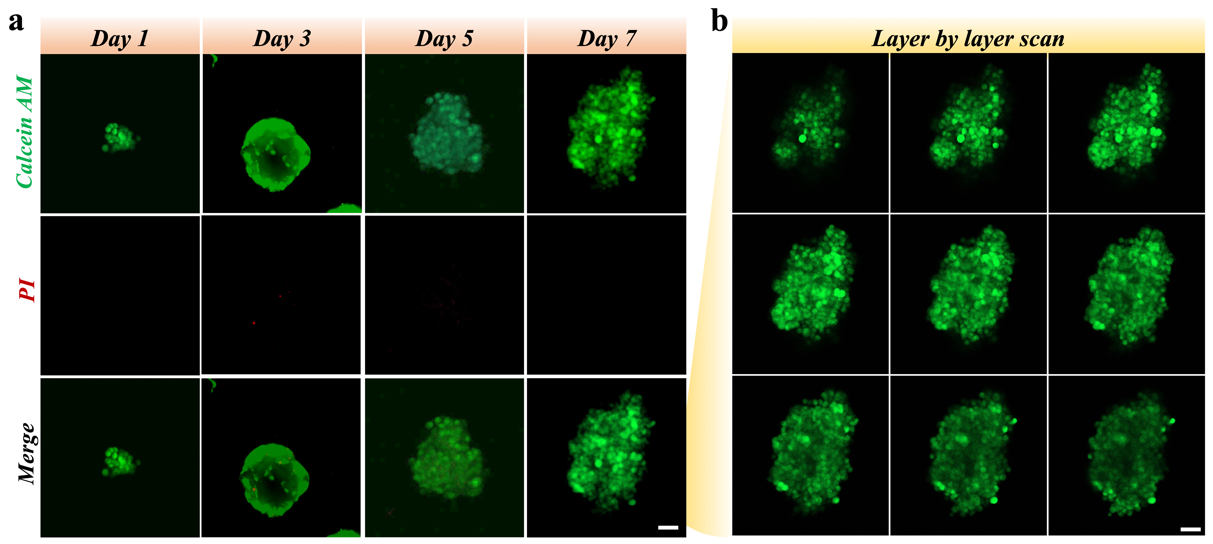
**

**Figure S3.** (a) The Calcein AM/PI staining showed that the encapsulated cells gradually proliferated in the core and formed spheroids. (b) The layer by layer staining of the spheroid measuring 250μm in size. The scale bar is 50 μm.


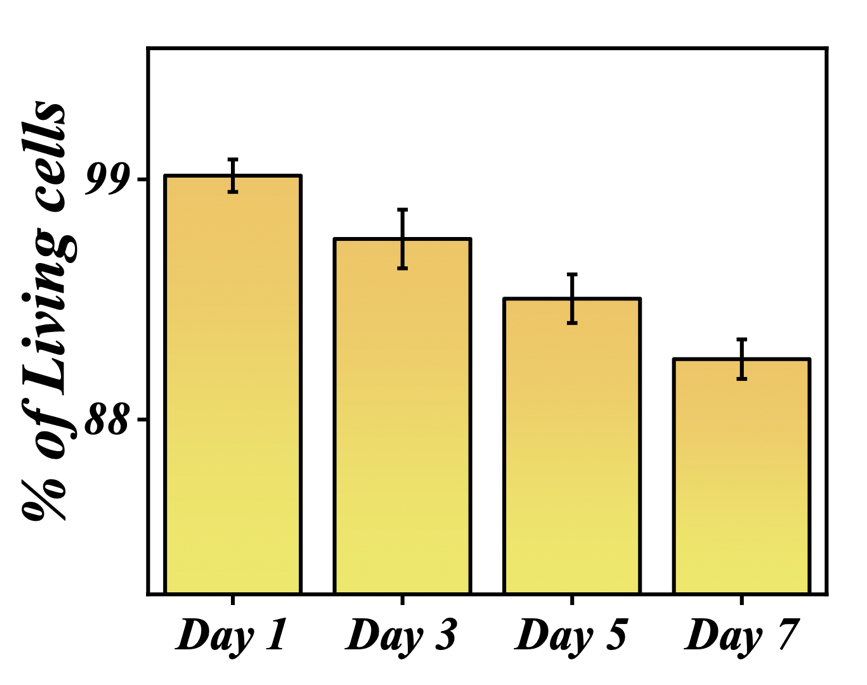


**Figure S4.** The quantification of living staining, the error bar represents standard deviation. All data are presented as means ± SE (n = 3).


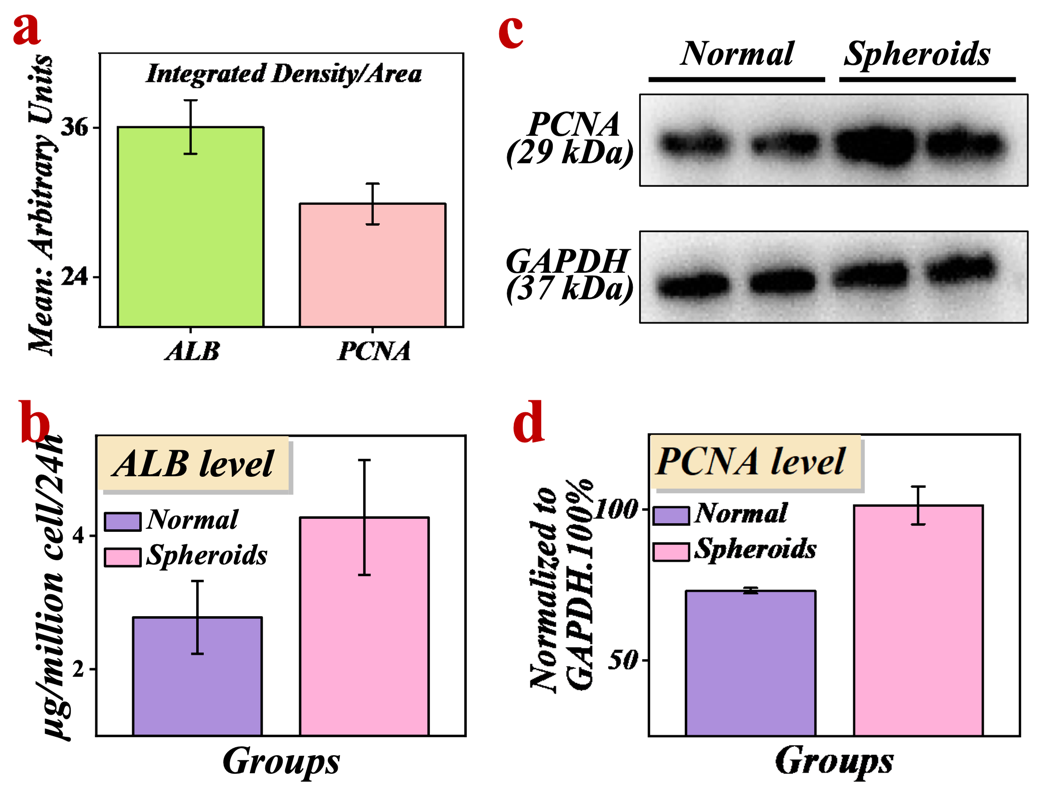


**Figure S5.** (a) The quantitative measurement of mean fluorescence intensity in ALB and PCNA staining in Figure 3b. (b-d) The examination of ALB and PCNA protein levels via ELISA (b) and Western blotting (c) analysis. (d) The quantification of Western blotting analysis. The cells in the normal group, cultured on a 2D plate, maintained an equivalent cell count to that of the spheroids group. All data are presented as means ± SE (n = 3).


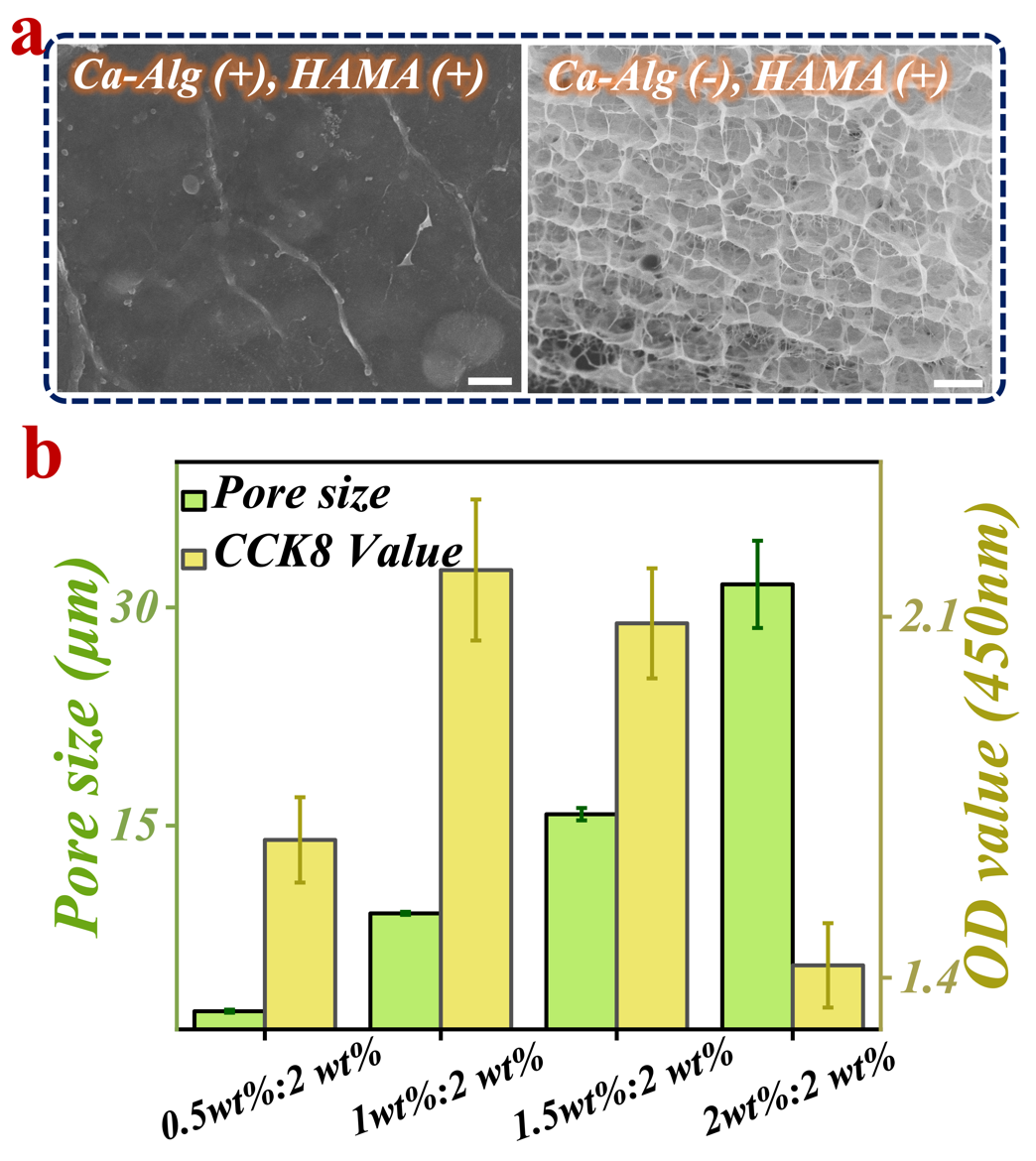


**Figure S6.** (a)The SEM images of Ca-Alg (+), HAMA (+) hydrogel and Ca-Alg (-), HAMA (+) hydrogel, the scale bar are 2 μm (left) and 10 μm (right). (b) The influence of Alg degradation at various weight percentages of Alg and HAMA on the pore size of hydrogels and encapsulated cell viability. All data are presented as means ± SE (n = 3).


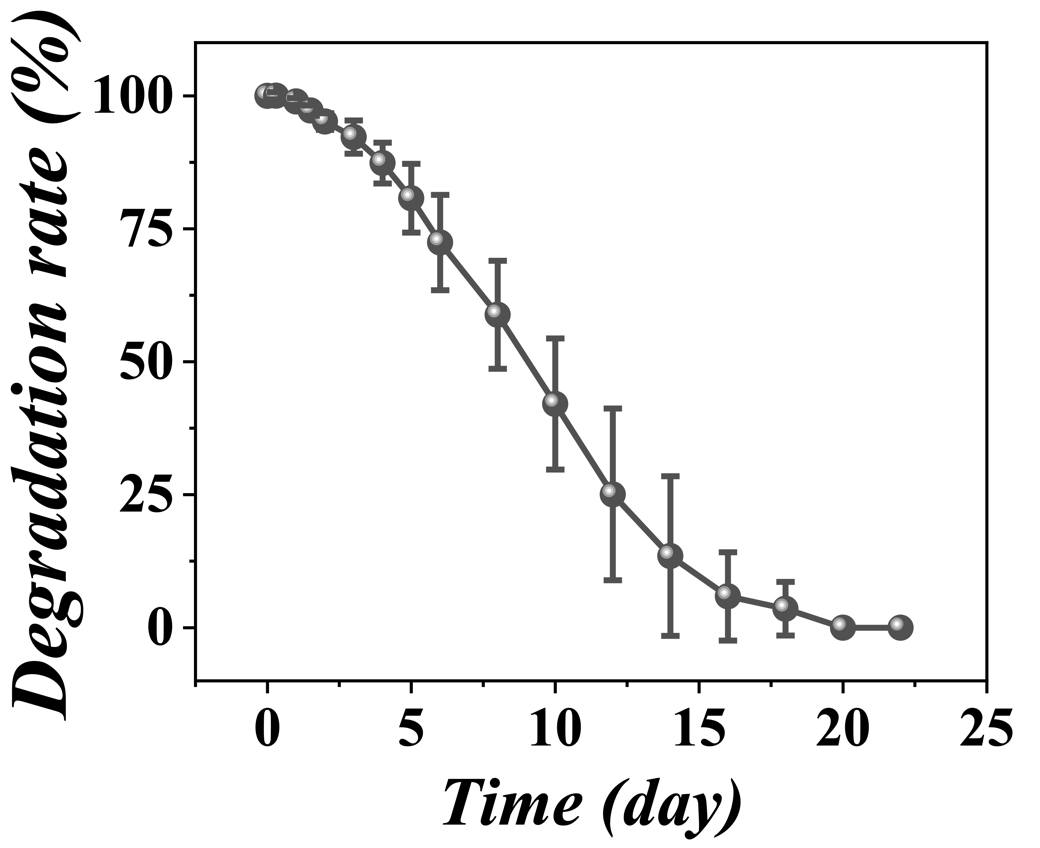


**Figure S7**. The degradation profile of microcapsules incubated in 20U/ml hyaluronidase solution was analyzed. In vitro degradation of Ca-Alg (-) HAMA (+) microcapsules was evaluated using enzyme-promoted degradation. Degradation rate (%) = (W_0_-W_t_)/W_0_ × 100%.


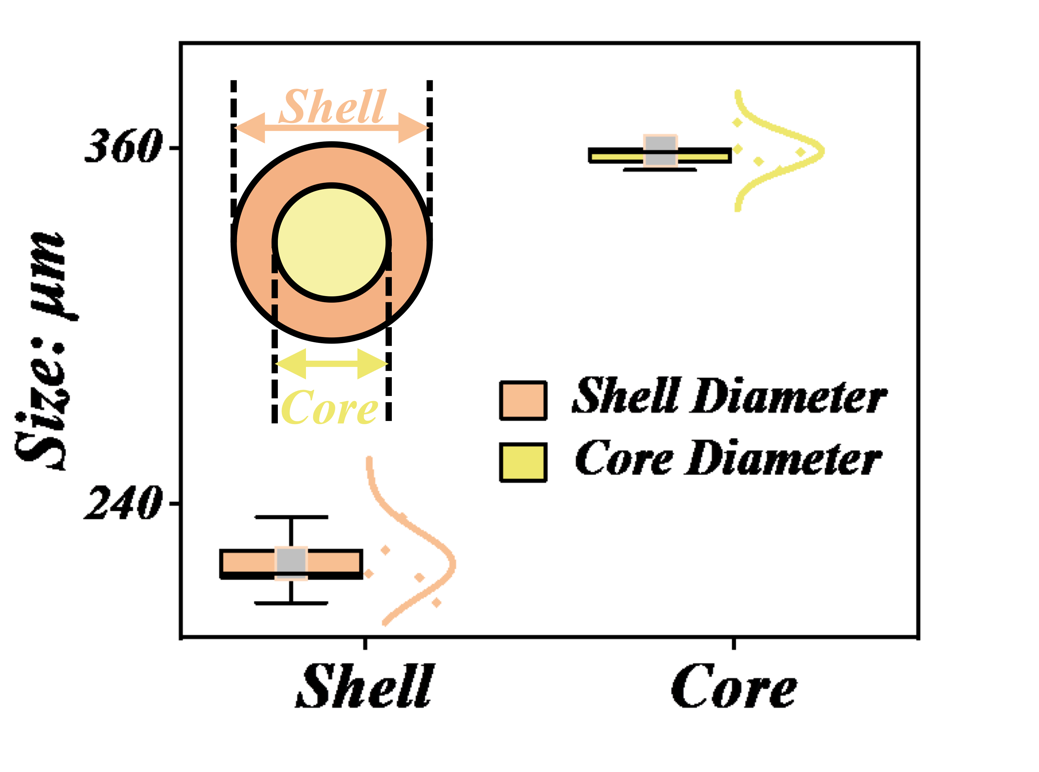


**Figure S8.** The dimensions of the core and shell within the iPSC-Hep spheroids that have formed within the microspheres. All data are presented as means ± SE (n = 5).

**
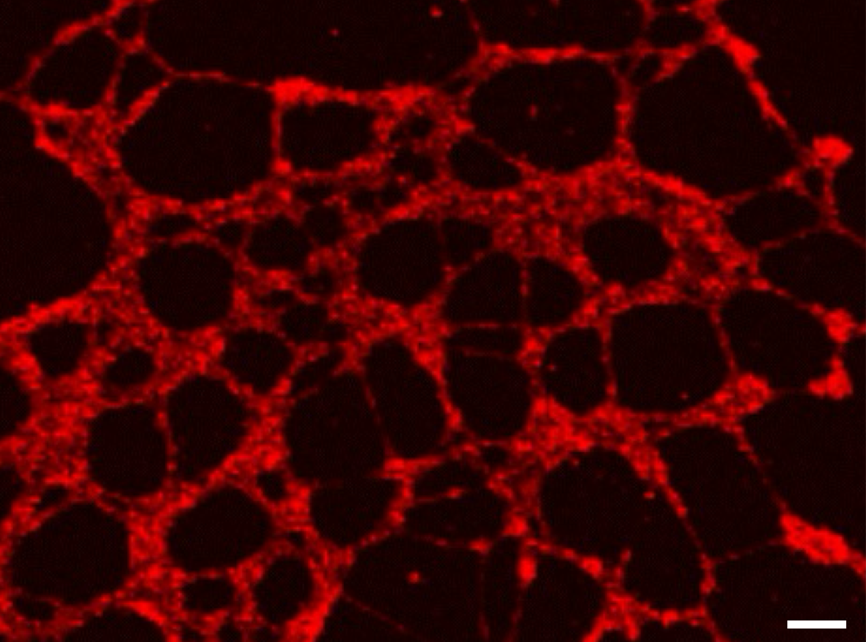
**

**Figure S9.** Laminin immunofluorescence staining on HUVECs encapsulated within the outer layer of microcapsules. Scale bars is 10 μm.


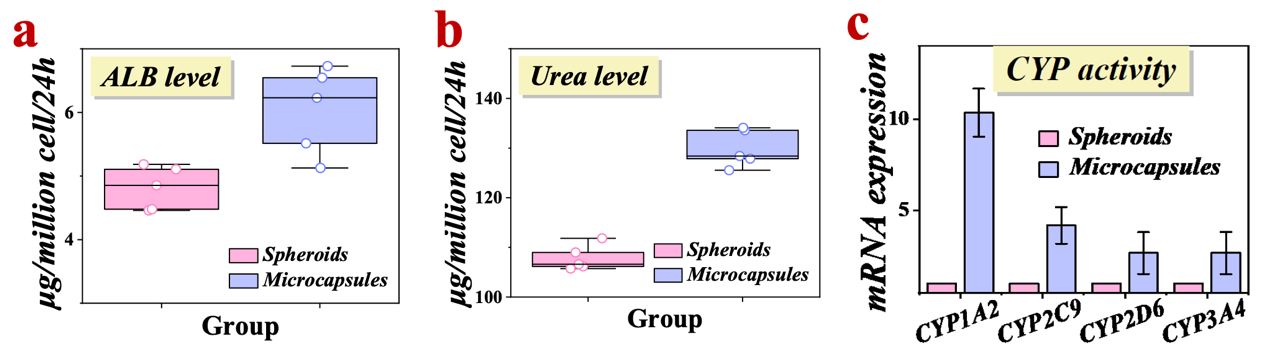


**Figure S10.** The assessment of ALB and Urea concentrations via ELISA (a, b) is complemented by the evaluation of CYP activity through real-time quantitative PCR (RT-qPCR) analysis (c). The spheroids group refered to the unadulterated hiPSC-hep spheroids, whereas microcapsules group denoted the vascularized hiPSC-hep spheroids. All data are presented as means ± SE (n = 5).

**Figure S11.** (a) Evaluation of VEGF levels in unvascularized hiPSC-hep spheroid microcapsules (referred to as the Spheroids group) and vascularized hiPSC-hep microcapsules (referred to as the Microcapsules group) using an ELISA. (b) Western blot analysis was employed to assess the levels of phosphorylated VEGFR2 and ZO-1 proteins in vascularized microcapsules containing hiPSC-hep spheroids, designated as Microcapsule groups (M1, M2, M3), and solely HUVECs-coated microcapsules devoid of hiPSC-hep spheroids, designated as HUVECs groups (H1, H2, H3). (c) The quantification of western blotting analysis. All data are presented as means ± SE (n = 3).


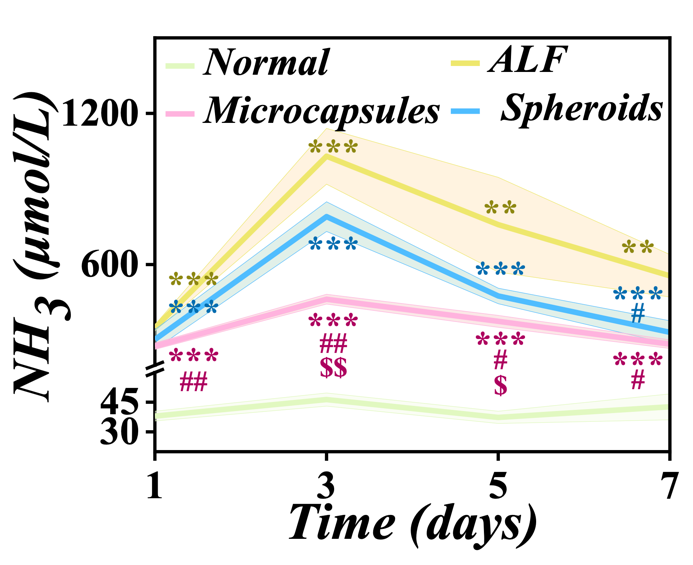


**Figure S12.** Evaluation of the NH_3_ level of ALF rats from different groups.

**
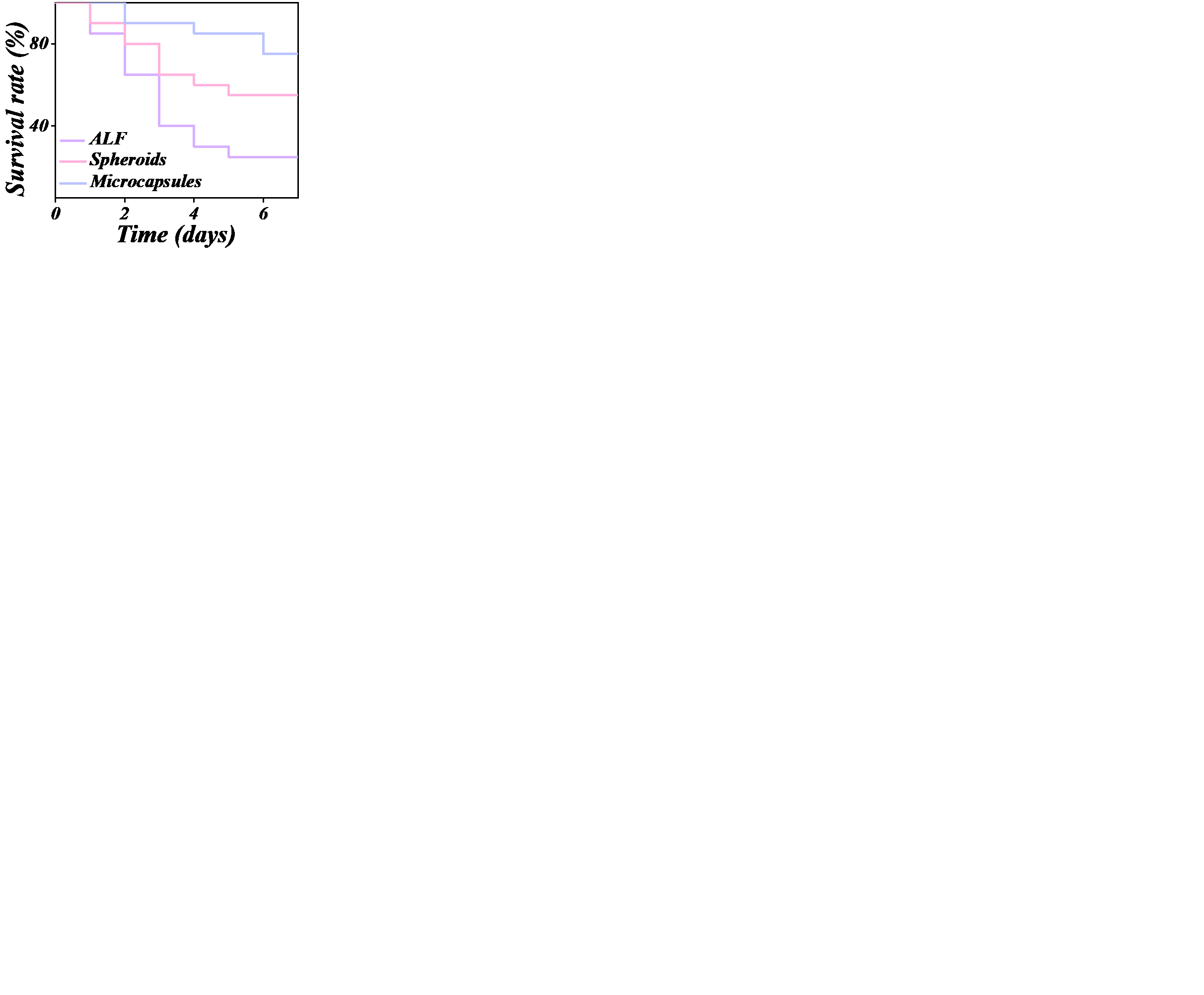
**

**Figure S13.** The survival rate of SD rats in three groups (n = 20).


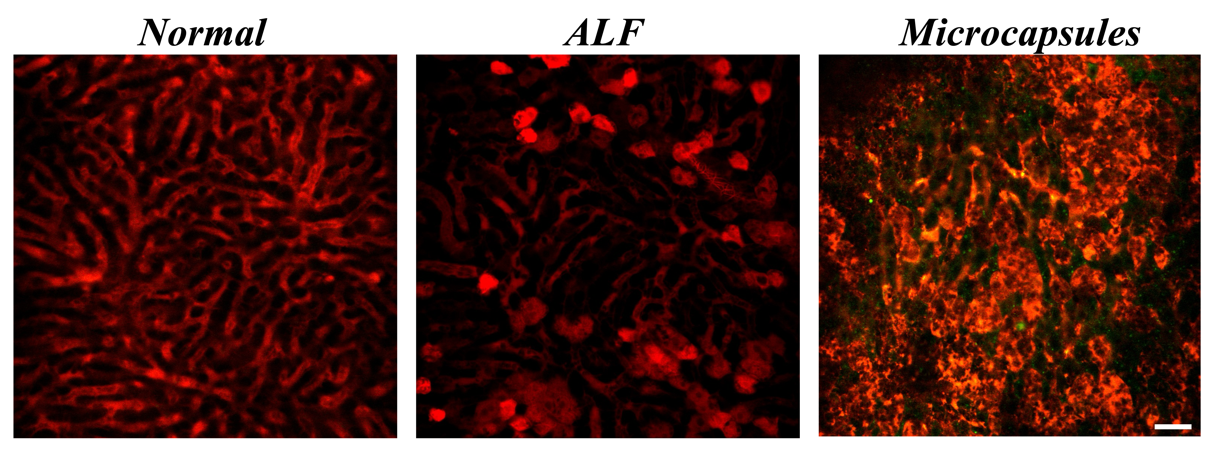


**Figure S14.** The IVIM's All-in-One Intravital Microscopy System demonstrated the blood flow (pseudocolored red using Evans Blue staining) in normal, ALF, and ALF rats following transplantation of vascularized microcapsules (labeled as Normal, ALF, Microcapsules group, respectively). Furthermore, HUVECs on the transplanted microcapsules are marked with GFP. The scale bar corresponds to 50μm.


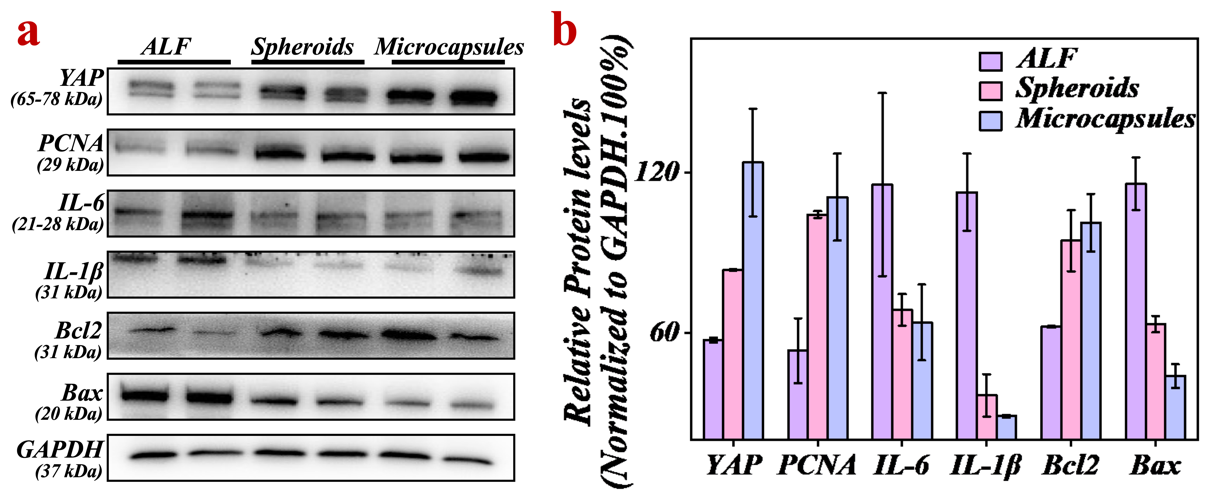


**Figure S15.** (a)Analysis of regenerative (YAP, PCNA), apoptosis-related proteins (Bcl2, Bax), and inflammatory factors (IL-6, IL-1β) via western blotting. (b) Quantitative interpretation of western blot findings. All data are presented as means ± SE (n = 3).


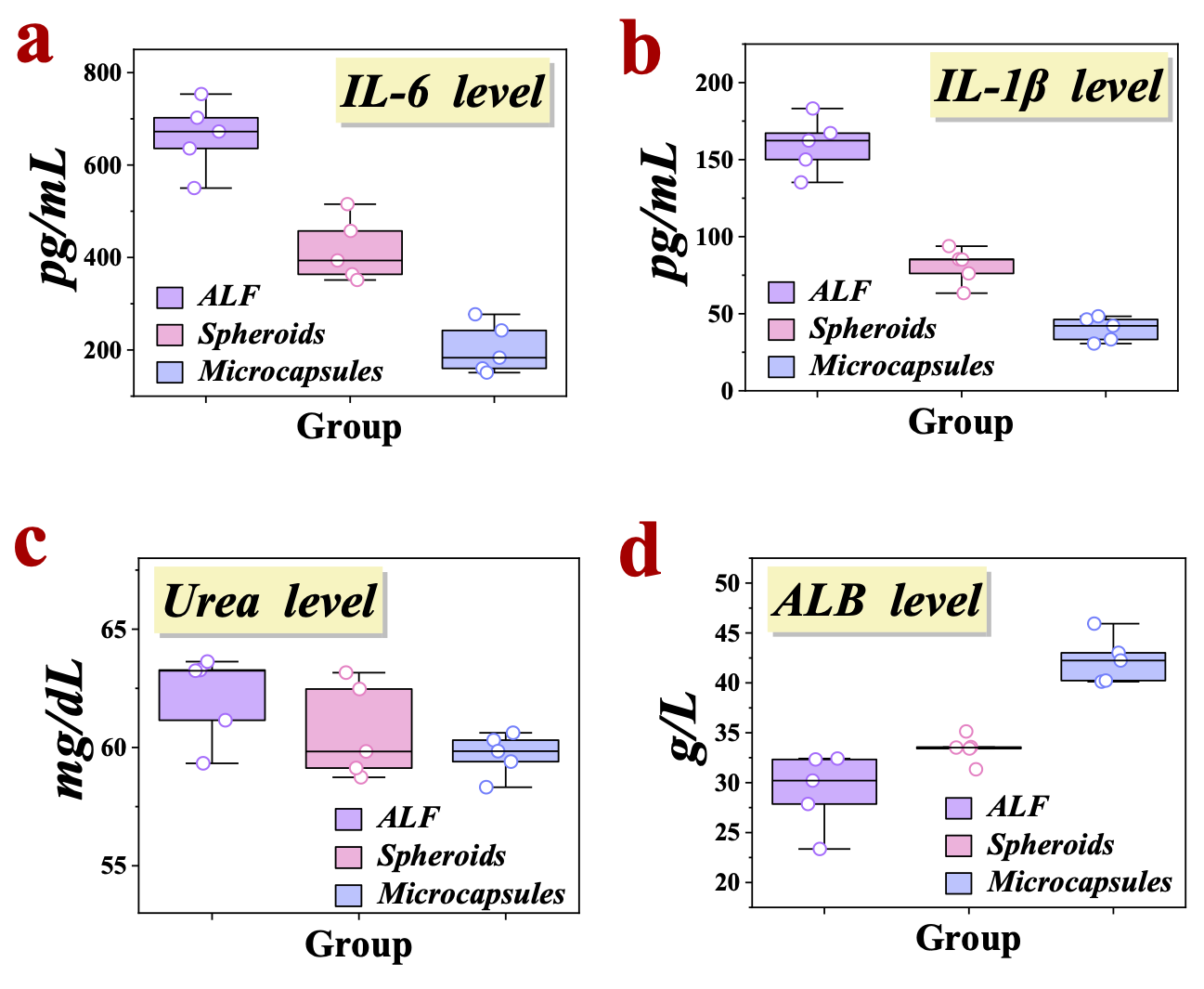


**Figure S16.** (a-d) Examination of IL-6, IL-1β, Urea, and ALB concentrations employing ELISA. All data are presented as means ± SE (n = 5).


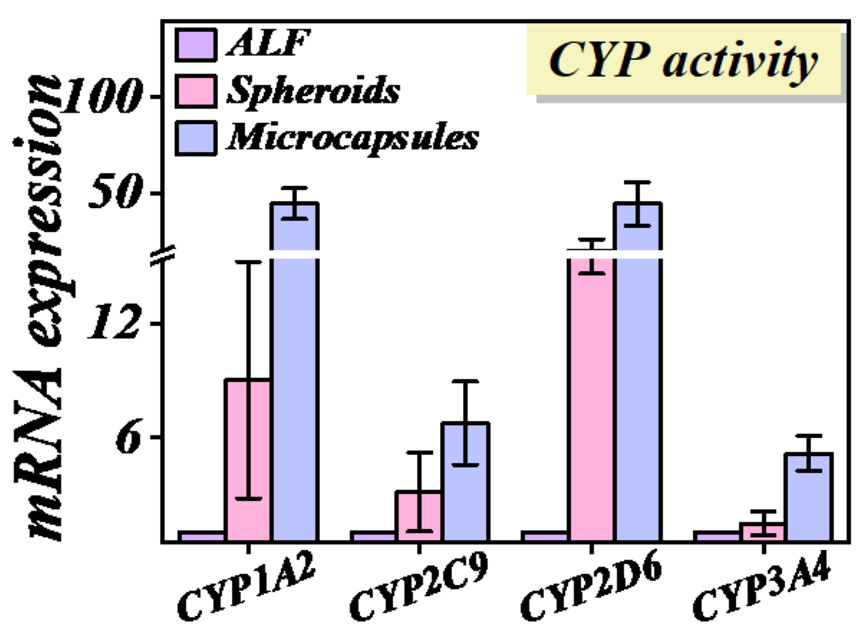


**Figure S17.** Assessment of CYP activity via real-time quantitative PCR (RT-qPCR) analysis. All data are presented as means ± SE (n = 5).


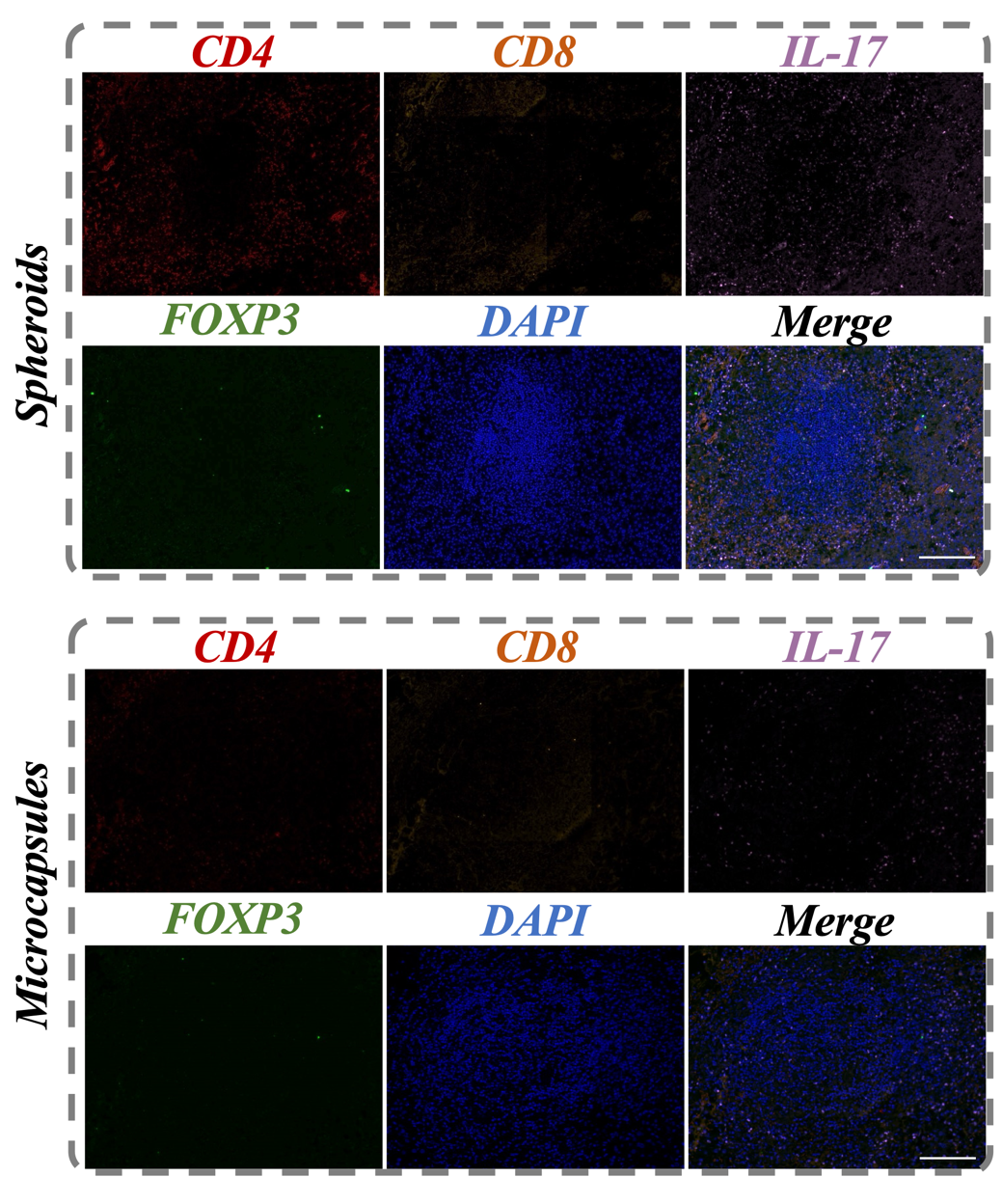


**Figure S18.** Different staining conditions of CD4, CD8, IL-17, and FOXP3 were assessed in liver tissue sections from each group using immunohistochemistry. The scale bar corresponds to 100 μm.
